# Cracking the code of health security: unveiling the balanced indices through rank-ordered effect analysis

**DOI:** 10.1186/s12913-023-10503-w

**Published:** 2024-01-04

**Authors:** Jianping Zhu, Qi Wu, Shiqi Zhang, Boliang Song, Weiwei Wang

**Affiliations:** 1 School of Management, Xiamen University, Xiamen, China; 2 The Second Affiliated Hospital of Xiamen Medical College, Xiamen, China; 3 Data-Mining Research Center, Xiamen University, Xiamen, China

**Keywords:** Health security, Balanced indices, Health infrastructure, Emergency preparedness and response, Cross-country comparisons

## Abstract

**Background:**

Health security is a critical issue which involves multiple dimensions. It has received increasing attention in recent years, especially in China. In order to improve the national health level, China has made many efforts, such as the “Healthy China 2030” plan proposed several years ago. However, due to the complexity of its national conditions and the difficulty of index design, the results of these efforts are not significant. Therefore, it is necessary to construct a new measurement index system.

**Methods:**

Based on the questionnaire of “Health China 2030”, we have collected a total of 3,000 participants from all 31 provinces, autonomous regions, and municipalities in China. We used statistical methods such as multiple correspondence analysis and rank-ordered effect analysis to process the data. The balance index is constructed by a series of actions such as weight division, order calculation and ranking.

**Results:**

Through multiple correspondence analysis, we can find that there was a close relation in the correspondence space between the satisfaction degrees 1, 2, and 3, while a far distance from satisfaction degrees 4 and 5. There were four positive and four negative indices separately based on the average expected level and four clusters after ordinal rank cluster analysis. Generally speaking, there are no prominent discrepancies across gender and residential areas.

**Conclusions:**

We created and examined balanced indicators for health security in China based on the “Health China 2030” questionnaire. The findings of this study give insight into the overall situation of health security in China and indicate opportunities for improvement.

**Supplementary Information:**

The online version contains supplementary material available at 10.1186/s12913-023-10503-w.

## Introduction

Health security is a critical issue, and it has become even more critical in recent years as a result of the outbreak of various infectious diseases such as SARS, Ebola, and COVID-19. China, which is one of the world’s most populous countries, has a large and diverse population and faces significant challenges in maintaining health security. Over a period of rapid economic growth, China’s reputation for health has been slipping. China’s health inequalities are increasing, gains slowing, and public dissatisfaction mounting [1]. In response to these challenges, the Chinese government has implemented a number of policies and programs aimed at improving the health of its people. The “Healthy China 2030” plan, which aims to promote the health and well-being of the Chinese people, is one of the most significant initiatives in this regard [2]. The National Health Commission (NHC) of China published the “Healthy China 2030”  (HC2030) plan in 2016, outlining a medium and long-term plan for national health development. As the first medium-long term strategic health planning at the national level, HC2030 undertakes the responsibility of safeguarding its people’s health by promoting healthy lifestyles, optimizing health services, improving health security, building a healthy environment, and developing health industries [3]. One of the primary aims of the plan is to develop a comprehensive health security system to preserve the health of the Chinese population [4].

Many existing studies have constructed indicators for comprehensive health security systems, and the Global Health Security Index is a classic example. Internationally, the Global Health Security Index (GHSI) is the first comprehensive assessment and benchmarking of health security and related capabilities across the 195 countries. However, questions remain over the skew of indicators towards the priorities of high-income countries, the validity of some indicators, the scoring system and its weighting [5]. In other words, the existing classical indicator system cannot cover all national environments. Therefore, to accomplish the goals of the HC2030 plan, effective methodologies and indicators for assessing health security must be developed. The current study proposes the process of creating balanced indicators and analyses the performance of each indicator for health security in China [6].

Balanced indices are composite indicators that take several dimensions of a concept or occurrence into consideration. Balanced indices for health security can assist to represent the complex and multifaceted character of health security by taking into account multiple dimensions of health, such as health outcomes, health determinants, and health system performance. The development of balanced indices for health security in China can provide a complete and systematic strategy to measuring and monitoring the country’s progress in health security [7]. Moreover, citizen participation and engagement are critical components of every public health initiative, particularly those targeted at increasing health security. As a result, the current study stresses the engagement of multiple stakeholders in the development and analysis of balanced indices for health security in China, such as policymakers, health professionals, and community members [8]. Citizen engagement may assist guarantee that the indices represent the interests and viewpoints of various groups, as well as facilitate the execution of the “Healthy China 2030” strategy. Prior research has demonstrated that balanced indices may be useful instruments for assessing and monitoring health security. The Global Health Security Index (GHSI), for example, is a composite indicator that assesses nations’ ability to prevent, identify, and respond to infectious disease threats [9]. Our study expands on earlier research by suggesting the development and analysis of balanced indicators for health security in China. The suggested indices can give a more personalized and accurate evaluation of health security in China by taking into consideration the country’s distinct context and problems. Furthermore, involving multiple stakeholders in the creation and research process can improve the indices’ relevance and application to the Chinese environment. In summary, the comprehensive index is a possible way to solve the problems of the current health security measurement system. Among them, the balance index as a representative is considered by us to further deepen the construction.

To construct a balanced index system, this paper describes the creation of a quantified measure of each domain in health security using the ordered-rank effect analysis, probes the discrepancies in health security across gender and residential areas, aims to provide evidence on health security equitability and practical implications for the public in China. Our study aims to contribute to the development of effective strategies and indicators for measuring and monitoring health security in China. By constructing and analyzing balanced indices based on the questionnaire “Health China 2030,” the study can provide a comprehensive and systematic approach to assess the progress of health security in the country. The involvement of various stakeholders can ensure that the indices reflect the priorities and perspectives of different groups and promote the implementation of the “Healthy China 2030” plan. The proposed indices can also serve as a reference for other countries facing similar challenges in maintaining health security.

The following is how the paper is structured. The next part examines the literature on health security and discusses the many indices that have been devised to assess health security in nations throughout the world. Then, we present our technique for developing balanced indices for health security in China, including the indicator selection and weighting of the various components of health security. Following that, we share our findings and examine the benefits and drawbacks of China’s health security system. Finally, we explore the ramifications of our findings as well as future research options.

## Literature review

### Health security in China

Health security has been a top priority for the Chinese government, especially since the SARS outbreak in 2003 and the ongoing COVID-19 pandemic [10]. The Chinese government has been implementing various policies and initiatives to improve the health security, such as the “Healthy China 2030” plan, which aims to improve the overall health of the population and enhance the country’s health security [2]. For these policies, we did some collection integration as shown in the Table 1. From the table, we can see that the Chinese government has done a lot to promote health security. However, despite these efforts, there are still challenges in achieving comprehensive health security in China. One major challenge is the uneven distribution of healthcare resources between rural and urban areas, with a consensus that rural areas often confronted with a shortage of healthcare professionals and inadequate medical facilities [11]. Another challenge is the lack of coordination between different government agencies and departments involved in health security [12], leading to inefficiencies and gaps in the system.

**Table 1:** Summary of China’s long-term health policy from 2009 to now

Policy/Program	Policy Publisher(s)	Objectives
Healthy China 2020 program (2009)	Ministry of Health (Now NHC)	1. By 2010, establish a preliminary framework for a basic healthcare system covering urban and rural residents, placing China among the nations implementing universal basic healthcare.
		2. By 2015, advance the level of medical and healthcare services, propelling China into the forefront of developing countries
		3. By 2020, maintain China's position at the forefront of developing nations, with urban and rural areas in the eastern region and some in the central and western regions approaching or reaching the levels seen in moderately developed countries.
Healthy China 2030 Planning Outline (2016)	Central Committee of the Chinese Communist Party and State Council of the People’s Republic of China (PRC)	1. By 2020, establish a China-specific basic medical and health system covering both urban and rural residents, elevate health literacy continuously, perfect and streamline an efficient health service system, ensuring universal access to basic medical and health services, and basic sports and fitness services. Form the foundation of a rich and well-structured health industry system, positioning major health indicators among the forefront of middle to high-income countries.
		2. By 2030, further refine the institutional system promoting national health, ensuring coordinated development in the health sector, widespread adoption of healthy lifestyles, continuous improvement in health service quality, and elevated levels of health security. Witness a thriving development in the health industry, achieving basic health equity, and major health indicators reaching the level of high-income countries. By 2050, construct a health nation compatible with the socialist modernization of the country.
HCI (2019–2030) Action plan for Healthy China 2030 policy	State Council of the PRC	1. Health Knowledge Promotion Initiative
		2. Balanced Diet Initiative
		3. National Fitness Campaign
		4. Anti-Smoking Initiative
		5. Mental Health Promotion Initiative
		6. Healthy Environment Promotion Initiative
		7. Maternal and Child Health Promotion Initiative
		8. Health Promotion in Primary and Secondary Schools Initiative
		9. Occupational Health Protection Initiative
		10. Elderly Health Promotion Initiative
		11. Cardiovascular Disease Prevention and Control Initiative
		12. Cancer Prevention and Control Initiative
		13. Chronic Respiratory Disease Prevention and Control Initiative
		14. Diabetes Prevention and Control Initiative
		15. Infectious and Endemic Disease Prevention and Control Initiative

To address these challenges, the Chinese government has been promoting the concept of “balanced development”, which involves ensuring equitable distribution of healthcare resources and improving coordination between different stakeholders [13]. This concept has been reflected in some policy documents, such as the “Healthy China 2030” plan, which emphasizes the importance of promoting “balanced development” in the health sector. Balanced development in the health sector involves various aspects, including improving the distribution of healthcare resources, strengthening the capacity of healthcare institutions, and enhancing coordination between different stakeholders. For example, the government has been implementing policies to encourage healthcare professionals to work in rural areas and providing financial incentives for healthcare institutions to improve their services [14].

Overall, while there have been significant improvements in health security in China, there is still room for further progress, particularly in achieving a more balanced and equitable healthcare system [15]. The concept of balanced development in the health sector provides a framework for addressing the challenges facing the Chinese healthcare system and improving health security for all citizens.

### Balanced indices for health security

Health security is a multi-dimensional concept that encompasses the prevention, detection, and response to health threats. The equitable distribution of health security is essential for achieving social welfare and economic progress [16]. But there are many confusions and distrust of definitions of it due to its substance as a multi-dimensional problem [17]. Therefore, measuring the synchronization of different fields within the health security system has been a challenge.

One way to address this challenge is through the use of balanced indices, which have been widely used in other fields to measure progress towards multiple goals simultaneously [18]. In the context of health security, balanced indices can help to identify gaps in different areas of health security and guide policy development towards a more comprehensive and integrated approach [12]. However, the construction of balance index is not comprehensive, and some applications still have deviations. We can see this from some existing applications in health security. The GHSI, which evaluates nations’ capacity to prevent, identify, and respond to infectious disease epidemics, is one example of a balanced index for health security [8]. But, the GHSI has been chastised for focusing on biological threats while ignoring societal determinants of health security such as poverty, inequality, and governance [19]. This example confirms the previous inference. So, there is a need for further research and development of balanced indices that capture the full range of biological and social determinants of health security.

### Construction and analysis of indices for health security in China

One of the major challenges in constructing indices for health security in China is the need to account for the complex and diverse nature of the country’s healthcare system. China’s healthcare system is highly decentralized, with different regions having different healthcare systems and policies. This decentralization makes it difficult to develop a standardized approach to measuring health security. Additionally, the healthcare system in China is a mix of public and private providers, which further complicates the development of a unified index.

Another challenge is the vast geographical size of China, which leads to regional disparities in healthcare infrastructure and resources. Some regions have better healthcare infrastructure and resources than others, which can affect the health security of the population in those regions [20]. This means that a single index may not be able to accurately reflect the health security situation in different regions of China. Furthermore, the healthcare system in China is constantly evolving, with new policies and reforms being implemented regularly. This means that any index developed for measuring health security in China needs to be able to adapt to these changes and be updated regularly to ensure its accuracy and relevance.

To find a suitable index, we review the previous application, one of the earliest indices proposed for measuring health security in China was the “Global Health Security Index”. This index measures health security based on various indicators, including prevention, detection and reporting, and response to health emergencies [8]. It belongs to a kind of balance index and has a relatively complete dimension construction. However, some researchers have criticized the index for its limited applicability to the Chinese context, given the differences in healthcare systems and policies [18]. This means that a single balanced indicator cannot be applied to all health security environments. In different national backgrounds, the use of balance indicators should be adjusted accordingly.

The studies reviewed in this section highlight the importance of incorporating various indicators and methodologies in the construction and analysis of indices, as well as the need for continuous monitoring and evaluation of the performance of the healthcare system. In addition, the uniqueness of the national environment will also have a corresponding space in the subsequent discussion.

## Materials and methods

### Data sources

For construction of health security indices, we obtained data of the status about health security issues in China from the National Survey of Health Security Issues (see appendices), which was conducted through an anonymous online questionnaire from 2020 to 2021 in 31 administrative regions across China. The principles of credibility, effectiveness and moderation were adopted in the questionnaire design [21, 22]. Following the sequence of questionnaire title, questionnaire description, informed consent, demographic information, and topic themes, a total of 24 questions were included at last. Given the importance to comply with universality, we strived to be concise, comprehensive, and accurate without any inducement or suggestions in question statements [23]. The questionnaire was finalized after several rounds of testing, discussion, modification and improvement. The study contents of interest are anchored at the target of HC2030 from viewpoints of health security system and health security service. As for health security system, medical care system, public health service system, and contingency response to public health emergencies system were considered, both the most fundamental health security issues globally; while environment stewardship, medical care convenience, surrounding public fitness facilities, rationality of the medical services price, and food & drug safety guarantees were considered as health security service. The main items of questionnaire are shown in Table 2.

**Table 2:** Questionnaire design

Dimensions	Items
Cognition of health security	Extent of understanding Healthy China 2030
Health security system	Contingency response system of public health emergencies, Basic medical care system, and Basic public health services system
Health security service	Status of environmental stewardship, Food and drug safety guarantees,
	Surrounding public fitness facilities, Medical care convenience, and Rationality of medical service prices

We designed the sample frame according to China Statistic Yearbook-2019 complied by National Bureau of Statistics of China [24], and issued questionnaires proportionally. However, as this research was conducted by means of web survey, the Internet users’ structure, nonresponse error, web behaviour differences, urbanisation advances, and socioeconomic influences jointly caused sample distribution deviation from that expected [25–27]. Nonresponse error arises through the fact that not all individuals included in the sample are willing or able to complete the survey [28]. For example, the seniors (65 years and above) accounted for 11.9% of the population, while the proportion of elderly Internet users is relatively low [29], partly because whether the seniors are willing to access the Internet depends on the existence of sufficient external support, moreover, the willingness of the elderly to use the Internet mainly for socialising, recreation and information, while less for online actions (including answer questionnaire) and business transactions [30]. With regard to the size of urban and rural Internet users, urban internet users are more than twice as many as rural internet users, with the ratio of 69.6% versus 30.4% at the beginning of 2020 [31]. As this study was designed to get an overview of health security nationwide, the representativeness of area region appears prominent, the population size, per capita disposal income, and financial budget (e.g., public security, social security and employment, and health services) all regarded as considerations in provinces distribution. Based on sampling method only, we considered population size individually and performed sample weighting accordingly to get an overall performance on health security prior to carrying on the rank-ordered effect analysis.

**Fig. 1:**
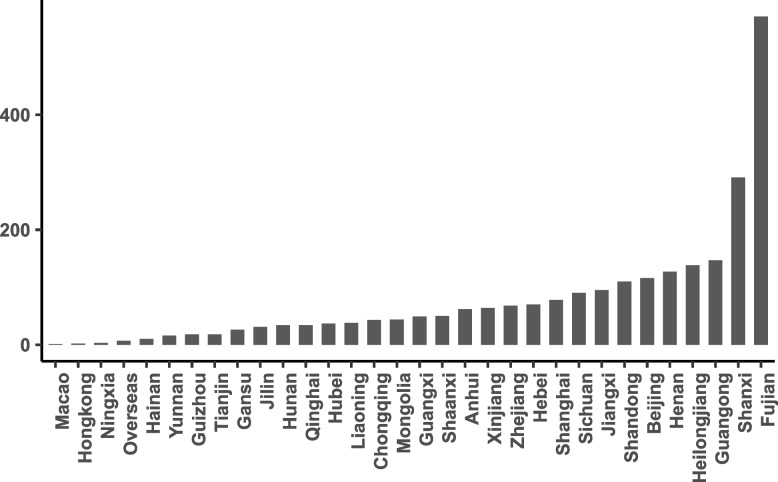
Spatial distribution of the participants

After an exclusion of irregular sample, a total of 2488 valid questionnaires were collected, with 80 surveys per administrative region on average. Survey results were weighted in order to represent an unbiased reflection of health security issues. Inclusion criteria include all the population aged 20 years or older and concerned with this topic, while those who did not answer questions were excluded considering the reliability of this study. A spatial distribution of the participants (Fig. 1) and a summary of demographic features (Table 3) were exhibited in the following. In order to ensure the validity and reliability of the study, we calculated the Cronbach’s alpha value and KMO statistic. The Cronbach’s alpha for all questions was 0.719, and the KMO statistic was 0.759, which was statistically significant at the 0.001 level, indicating that the questionnaire has a good structural validity.

**Table 3:** Summary of demographic information of the participants

Variables	Value	Sample size	Sampling distribution
Age	< 20 years	121	4.86%
	20–34.9 years	1218	48.95%
	35–44.9 years	614	24.68%
	45–59.9 years	501	20.14%
	> 60 years	34	1.37%
Gender	Male	1100	44.21%
	Female	1388	55.79%
Area	Urban	1941	78.01%
	Rural	547	21.90%
Occupation	Personnel of party and government offices, social organizations, enterprises and institutions	510	20.5%
	Teachers and professional technicians	750	30.14%
	Business and service personnel	389	15.64%
	Production operator	48	1.93%
	Others (students, soldiers, etc.)	791	31.79%
Educational background	Primary school	7	0.28%
	Junior high school	50	2.01%
	High school or technical school	85	3.42%
	Junior college or bachelor’s	1077	43.29%
	Master’s degree & above	1269	51%
Health status	Very well	948	38.1%
	Fine	1060	42.6%
	General	405	16.28%
	In poor shape	16	0.64%
	Suffering from chronic diseases	59	2.37%

### Methods

#### Multiple correspondence analysis

Multiple Correspondence Analysis (MCA) is a statistical technique used for analysing the relationships between categorical variables [32]. As an extension of correspondence analysis which is primarily designed for two-way contingency tables [33], it allows to analyse the pattern of relationships of several categorical dependent variables and is widely accepted as the multivariate generalization of simple (bivariate) correspondence analysis [31]. The main goal of MCA is to represent the relationships among categories of different variables in a lower-dimensional space. This is achieved by creating a graphical representation that highlights the patterns and associations between the categories. It is particularly useful when dealing with datasets where multiple categorical variables are present and researchers want to explore the interdependence or associations among these variables [34]. In our research context, the construction of balance index needs to consider the influence of different categories of factor rank on various categories of health security items. The application of multiple response analysis method can more clearly show the influence path and the influence difference among different categories.

#### Rank-ordered effect analysis

Rank-ordered effect analysis is commonly used for dealing with categorical variables, and the balanced indices were built accordingly following the steps below [35]:

1. Sample weighting. In order to balance the sample distribution across provinces better, we performed sample weighting with consideration of population size in each province during sample period at the beginning of rank-ordered effect analysis. Let ${p}_{f}^{*}$ be the population ratio of province $f$, $f=\mathrm{1,2},\cdots ,31$, and ${p}_{f}$ be the sample ratio of province $f$ in this survey. We define ${q}_{f}= {p}_{f}^{*}\times {p}_{f}$ and normalize it as

$$\begin{array}{cc}w_f=\frac{q_f}{\sum_{f=1}^{31}q_f}&f=1,2,\cdots,31\end{array}$$

Based on the initial sample size ($N$ = 2488), the weighted sample size of province $f$ could be calculated as:

$$\begin{array}{cc}n_f^\ast=w_f\times N&f=1,2,\cdots,31\end{array}$$

2. Build an ordered table data. Take five different levels of *X* marked as {${X}_{j}, j=\mathrm{1,2},\dots ,5$}, represent different levels of satisfaction ranked from the lowest to highest. Take labels *Y* representing the eight items of health security marked as ${Y}_{1},{Y}_{2},\dots ,{Y}_{8}$, with observed frequencies marked as $\left\{{n}_{ij},i=\mathrm{1,2},\cdots ,8,j=\mathrm{1,2},\cdots ,5\right\}$, as shown in Table 4.

3. Calculate the mean rank of the ordered factors *X*. We described ordered factors as ${X}_{j}=({n}_{1j},{n}_{2j},\cdots ,{n}_{8j}{)}{\prime},j=\mathrm{1,2},\cdots ,5$, with the intervals $[{\sum }_{t=1}^{j-1}{n}_{.t}+1,{\sum }_{t=1}^{j}{n}_{.t}]$ as rank intervals, and ${\overline{R}}_{j}$ as the average rank of intervals, where ${\overline{R}}_{j}=\frac{\left(2{\sum }_{t=1}^{j=1}{n}_{.t}+{n}_{.j}+1\right)}{2}$, $j=\mathrm{1,2},\cdots ,5$.

4. Calculate the rank of items *Y*. The sum of the ranks of level $i$ among items *Y* relative to ordered factors *X* was denoted as $R_i,\;i\;=\;1,2,\;\cdots\;,\;8.$ 

$$\begin{array}{cc}R_i=\sum\limits_{j=1}^5{\overline R}_j\times n_{ij}&i=1,2,\cdots,8\end{array}$$

*R*_i_ was the rank effect of level *i* in *Y* affected by the ordered factors *X*. The rank weight was calculated through normalization,

$$\begin{array}{cc}\omega_i=\frac{R_i}{\sum_{i=1}^8R_i},&i=1,2,\cdots,8\end{array}$$

5. Calculate balanced indices. Each index was regarded as part of the whole system, with the mean value regarded as the expected level on average, and the balanced indices $\mu_i$ were established on the basis of the rank weight $\omega_i$ 

$$\begin{array}{cc}\mu_i=\left[\frac{\omega_i}{\left(\frac18\right)}\right]\times100\%&i=1,2,\cdots,8\end{array}$$

**Table 4:** Unidirectional ordered table

	Not satisfied at all (*X*_1_)	Dissatisfied (*X*_2_)	Generally satisfied (*X*_3_)	Almost satisfied (*X*_4_)	Very satisfied (*X*_5_)
Contingency response to public health emergencies (*Y*_1_)	18.39	94.78	392.64	1185.57	798.62
Medical care system (*Y*_2_)	19.88	187.83	776.40	1270.66	235.24
Environment stewardship (*Y*_3_)	47.36	236.26	795.40	1157.67	253.31
Basic public health services (*Y*_4_)	34.00	218.00	817.00	1228.00	193.00
Medical care convenience (*Y*_5_)	53.43	315.26	761.18	1135.06	225.07
Surrounding public fitness facilities (*Y*_6_)	48.09	315.08	831.25	1097.95	197.63
Rationality of the medical services price (*Y*_7_)	101.14	468.72	894.76	929.17	96.21
Food and drug safety guarantees (*Y*_8_)	115.01	462.30	919.82	855.42	137.45

Those scored higher than the average expected level (*µ*_*i*_ > 100) are positive, otherwise the indices are negative (*µ*_*i*_ < 100). The higher the value is, the better the performance of balanced indices is. As in one system, interrelationships exist between one another, just like playing seesaw. The balanced indices can be used to measure the performance of each index from systematic view.

#### Ordinal rank cluster analysis

The goal of clustering is to maximize the similarity within intra-cluster and minimize the similarity between inter-cluster. In this paper, we present an ordinal rank cluster algorithm, with the main steps involved as follows [36]:

1. Sequence eight indices *µ*_*i*_(i= 1,2, …, 8) from the highest to the lowest and calculate the adjacent distances of *d*_*i,i *+1_ = *µ*_*i*+1_ **-*** µ*i (*i* = 1,2, …,7).
2. Set appropriate threshold *α*, split the directional chain whenever *d*_*i,i+*1_ *> *α.
3. Calculate the variances till reaching the aim of maximization of similarity within intra-cluster and minimization of similarity between inter-cluster.

## Results

### Multiple correspondence analysis

**Fig. 2:**
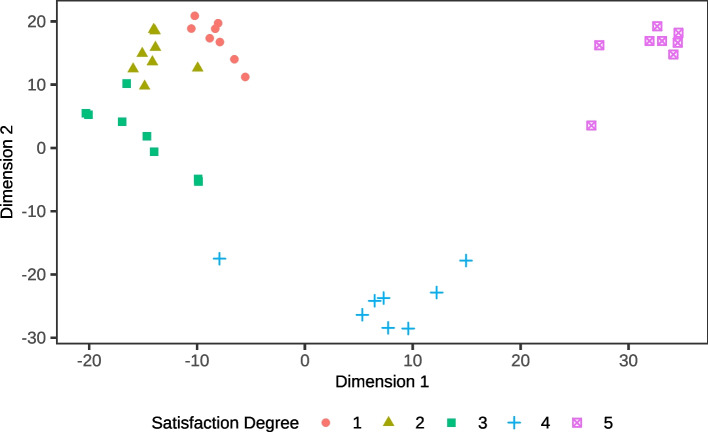
The correspondence of eight variables in five categories

In order to explore how closely the categories 1 to 5 of variables 1 to 8 are each other and how pairwise association reflected, we carried on multiple correspondence analysis. As shown in Fig. 2, there is an intuitionistic reflection of the pairwise association structure of the eight variables and it is possible to deduce how the eight variables correlated. We can find that there was a close relation in the correspondence space between the satisfaction degrees 1, 2, and 3, while a far distance from satisfaction degrees 4 and 5.

### Balanced indices and ordinal rank cluster analysis

**Fig. 3:**
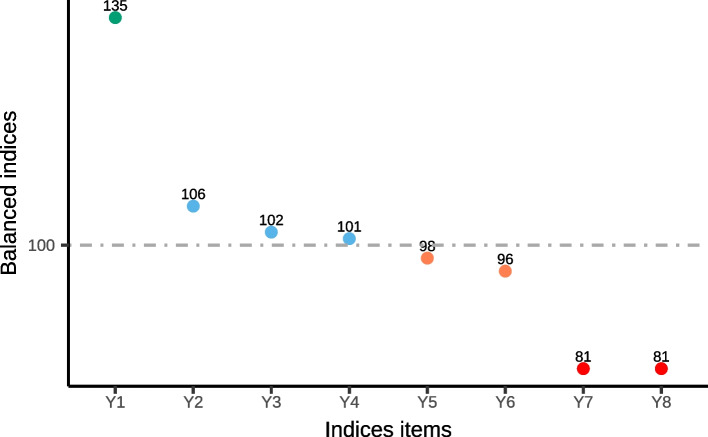
Distribution of eight indices for health security

The balanced indices in eight domains of health security were calculated according to rank-ordered effect analysis. As shown in Fig. 3, the eight indices can be divided into two groups based on the average expected level, those exceeding 100 denoted as positive indices, including the contingency response to public health emergencies (*Y*_1_), medical care system (*Y*_2_), environmental stewardship (*Y*_3_), and basic public health service system (*Y*_4_); and the others are negative ones, including medical care convenience (*Y*_5_), surrounding public fitness facilities (*Y*_6_), rationality of medical services prices (*Y*_7_), and food & drug safety guarantees (*Y*_8_). According to the values of eight indices, there were four positive and four negative indices separately based on the average expected level and four clusters after ordinal rank cluster analysis. Specially, contingency response to public health emergencies got an extraordinary excellent performance compared to others. As the indicator most influenced by COVID-19, we can conclude that Chinese people show a positive attitude toward Chinese government tackling with the epidemic. Four clusters formed according to the algorithm of clustering as Class 1 (*Y*_1_), Class 2 (*Y*_2,_ *Y*_3,_ *Y*_4_), Class 3 (*Y*_5,_ *Y*_6_), and Class 4 (*Y*_7,_ *Y*_8_), with inter-cluster variance (286.86) far greater than the intra-cluster variances (0.00, 7.00, 2.00, 0.00).

### Indices comparisons across gender and residential areas

**Fig. 4:**
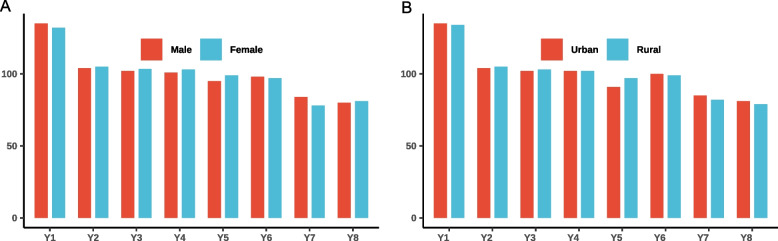
Comparisons of eight indices across gender (**A**) and residential areas (**B**)

In order to explore the equity of each index across gender and residential areas, we made a comparison of each indicator accordingly. Generally speaking, there are no prominent discrepancies across gender and residential areas. In detail, the indices of medical care system, public health service system, environment stewardship, and food & drug safety guarantees got a slightly higher score in males compared with females. As for the comparison of urban and rural areas, the indices of medical care system, public health service system, surrounding public fitness facilities, and the rationality of medical services price got a relatively higher score in urban residents than in rural residents. The intuitionistic reflection of comparisons was demonstrated in Fig. 4.

## Discussion

### Construction of health and safety system and service indicators under the Healthy China 2030 goal

This study anchored at the target of Health China 2030, which encompasses popularizing healthy life, optimizing the health service, improving health protection, and building healthy environment. In order to construct a framework applicable for such study, combining the essence of health security, which is really a comprehensive capability for country to guard the people from health threats, such as environmental changes, pollutant expansion, disease attack, and societal challenges, we decided to consider this issue from viewpoints of health security system and health security services, giving consideration to items that are widely focused and a total of eight indices included finally. Health security is composed of connected networks of health polices and health services, while the order, structure, harmony and alignment within and amongst systems can be interpreted as coherence, which always implies correlations, connectedness, consistency and efficient energy utilization. Every component is not only part of the whole system, but also can be seen as a whole itself [37]. 

After intuitionistic reflection of overall performance and pairwise association structure of the eight fields, the concern that the hierarchy of importance with category agents is not clear. The formulation of the agents’ importance hierarchy through a rank-ordering is easier (for the analysts) than that through a set of weights [38]. This article proposed a set of relatively simple and intuitive indices for assessing quality classification problem with multiple rank-ordered agents. When processing the unidirectional sequential data, the order or magnitude of each level of the ordered factors should be fully considered, and be treated as semi-quantitative indicators, thus get a comparison of impact magnitude between classes.

After the devastating impact of SARS in 2003, China launched an ambitious program to improve detection ability of new threats, strengthen response capacity, and report events more transparently. Enhanced facilities, state-of-the-art equipment, standard operating procedures, and objective assessment exercises have been part of the enhancements of Emergency Operations [39]. The time from the start of an outbreak to detection and response has decreased, as it has in many other countries that have strengthened disease detection capacity [40]. The confidence index fluctuates at major time points with the development of events, but it tends to be stable after February 24th, showing overall confidence in the fight against epidemic based on Bernstein’s model analysis [41]. From the distribution of the hot topics, the government response has been the top one since outbreak of the epidemic, and the Chinese people have maintained a rational and positive attitude in the face of the event [42].

### Significant progress in healthcare, environmental protection, and basic public health services

China has made significant strides in the health care system, environmental stewardship, and basic public health service system. These three indicators provide a comprehensive overview of China’s basic health security, encompassing the dimensions of basic health security, environment, and disease prevention. The high scores in these areas demonstrate that China has performed well in these domains, with values that exceed the average expected level. Specifically, the health care system in China has undergone significant improvements, with increased access to healthcare services and improved quality of care. The government has invested heavily in healthcare infrastructure, including the construction of new hospitals and clinics, and the upgrading of existing facilities. This has resulted in a significant increase in the number of healthcare professionals, including doctors, nurses, and other healthcare workers. In terms of environmental stewardship, China has made significant progress in reducing pollution and improving air and water quality. The government has implemented a range of policies and regulations to reduce emissions from industrial and transportation sources, and to promote the use of renewable energy sources [43]. These efforts have resulted in a significant reduction in air and water pollution, and have contributed to a healthier environment for all citizens. The basic public health service system in China has also undergone significant improvements, with increased access to preventive healthcare services and improved disease prevention measures. The government has implemented a range of programs and initiatives to promote public health, including vaccination campaigns, health education programs, and disease surveillance systems. These efforts have resulted in a significant reduction in the incidence of infectious diseases and other public health threats.

Despite the progress made in the health care system, environmental stewardship, and basic public health service system, there are still areas that require improvement. One of the main challenges is the convenience of health care services and public fitness facilities. The regionalization of health resources in China has led to disparities, with some regions having better access to healthcare services than others. This has resulted in a lack of balance and inadequacy in the development of healthcare services, which has not kept pace with the increasing health needs of the population. The contradiction between the unbalanced and inadequate development of health care services and the increasing multi-level and diversified health needs of the masses is very prominent. As the population grows and ages, the demand for healthcare services is increasing, and the needs of the population are becoming more diverse. However, the development of healthcare services has not been able to keep up with these changes, leading to a gap between the supply and demand of healthcare services.

To address the challenges in the health care system, environmental stewardship, and basic public health service system, the Chinese government has launched the HC2030 initiative. This initiative emphasizes the importance of physical fitness facilities for the entire population and aims to improve the convenience of health care services and public fitness facilities. The government has issued guidelines to improve public fitness facilities and is committed to making considerable efforts to enhance these services. This includes investing in the construction of new fitness facilities, upgrading existing facilities, and promoting physical fitness programs and activities. However, more needs to be done to address the regional disparities and improve the convenience of health care services and public fitness facilities. The government needs to ensure that healthcare services and fitness facilities are accessible to all citizens, regardless of their location or socio-economic status. This can be achieved by investing in infrastructure development, improving transportation networks, and providing financial support to disadvantaged communities. By addressing these challenges, the Chinese government can ensure the health and well-being of all citizens and create a more equitable and sustainable healthcare system. The HC2030 initiative is a positive step towards achieving this goal, but more needs to be done to fully address the challenges in the health care system, environmental stewardship, and basic public health service system.

### Urgent imperative for improvement in health service prices and food & drug safety in China

An urgent imperative improvement is needed in domains of rationality of health services prices and food & drug safety guarantees. The two indicators got the lowest score. It’s obvious the issue of high expense of health care is still a grim reality even after several healthcare reforms. Government financing as a proportion of total health expenditure decreased from almost 40% in the early 1980s to 18% in 2005, while out-of-pocket payments rose from 20% to more than 50% in the same period [44]. In an analysis of the national household health surveys in 1998 and 2003, Xu found that out-of-pocket payment for hospital care increased for all income groups in the 5 years between surveys [45]. Individually, those in poor economic conditions have to spend a much greater proportion of their income on health care than richer people do. In addition to the high cost of health care, there are also significant issues relating to food & drug safety in China. Some of the realities that exist in China include source pollution, weak foundation of the food industry, and gaps between food safety standards and those of international standards. These issues could pose a significant threat to public health.

To effectively address the issues of high health service prices and food & drug safety guarantees, the Chinese government adopts a comprehensive approach that encompasses multiple strategies. One such strategy is to increase government financing for healthcare, which can help alleviate the financial burden on individuals, particularly the poor, who are disproportionately affected by the high cost of health care. Additionally, the government must improve the regulation and enforcement of food safety standards to ensure that the food industry operates in a safe and responsible manner. This can be achieved by implementing stricter regulations, increasing inspections, and imposing harsher penalties for non-compliance. Furthermore, the government must address the root causes of source pollution, which is a significant contributor to food safety issues. This can be achieved by implementing policies that promote sustainable agricultural practices, investing in infrastructure to improve waste management, and enforcing environmental regulations. Implementing these strategies will require significant investment and a long-term commitment from the Chinese government. However, the benefits of such an approach are numerous. By addressing these issues, the government can improve the health and well-being of its citizens, reduce the burden of medical expenses on individuals, and create a more equitable and sustainable healthcare system. By ensuring the safety of the food supply, the government can also protect public health and prevent the spread of foodborne illnesses.

### Challenges and limitations in constructing balanced indices for health security in China

Given the fact that the construction and analysis of balanced indices for health security in China based on the questionnaire of “Health China 2030” is a valuable and timely endeavor that contributes to the understanding of the current state of health security in China. However, there are several limitations and challenges that need to be addressed in this research. One restriction of this study is the subjective character of the data collection questionnaire. The questionnaire was created using the opinions and judgments of public health specialists, which may not fully reflect the viewpoints and experiences of the general community. As a result, the findings of this study may not precisely reflect the real condition of health security in China. Another drawback of this study is the possibility of bias in the selection of indicators and weights utilized in the creation of the balanced indices. The researchers’ personal beliefs and prejudices may impact the selection of indicators and weights, affecting the validity and reliability of the results. As a result, it is critical to ensure that the indicators and weights are chosen using objective and transparent standards, and that the findings are subjected to thorough sensitivity analysis. Moreover, the possibility of geographical and socioeconomic variations in health security throughout China is a difficulty in this research. While the balanced indices created in this study try to describe China’s overall condition of health security, they may not fully reflect inequalities and variances among regions and socioeconomic categories. As a result, future study might include more granular data and analysis to capture the geographical and socioeconomic components of health security in China. Furthermore, the absence of longitudinal data to examine changes and trends in health security over time is a weakness of this study. While this study gives a glimpse of China’s present status of health security, it does not provide insight into changes and patterns in health security over time. As a result, future study might look into employing longitudinal data and analysis to analyze changes and trends in China’s health security.

## Conclusion

In conclusion, this study created and examined balanced indicators for health security in China based on the “Health China 2030” questionnaire. The findings of this study give insight into the overall situation of health security in China and indicate opportunities for improvement. This study’s balanced indices include a wide variety of health security characteristics, including health infrastructure, health workforce, illness prevention and control, disaster preparedness and response, and health outcomes. The balanced indices research shows that China has achieved substantial progress in enhancing its health security in recent years. Nonetheless, other sectors still need attention and investment, such as improved health infrastructure and emergency response skills. This study also emphasizes the significance of utilizing balanced indices to measure the condition of health security since they give a full and objective perspective of the many components of health security. Balanced indices may also be used to promote cross-country comparisons and benchmarking, which can assist uncover best practices and areas for development.

Notwithstanding the useful insights presented by this research, there are some limits and issues that require further investigation in the future. The subjective character of the questionnaire, the possibility of bias in the selection of variables and weights, geographical and socioeconomic variations in health security, and the lack of longitudinal data are all examples. Resolving these constraints and problems will assist assure the validity and trustworthiness of the data, as well as providing a more realistic picture of China’s health security.

Overall, the construction and analysis of balanced indicators for health security in China based on the “Health China 2030” questionnaire is a great addition to the area of public health. This study presents a complete and impartial assessment of the many aspects of health security in China, highlighting the areas that demand attention and investment. It is intended that the findings of this study would influence public health policy and decision-making, as well as contribute to the enhancement of health security not only in China, but also in other countries with similar experience.

### Supplementary Information

**Additional file 1. **Survey of health security issues statuses in China.

**Additional file 2: Table S1.** Survey of Health Security Issues in China - Overview.

## Data Availability

The data supporting the findings of this study are available in Table S1, which provides a comprehensive overview of the survey results on health security issues in China. Table S1 is included as a supplementary file accompanying this manuscript. Readers are encouraged to refer to Table S1 for detailed information on survey questions and responses.
