# Supplementary material for: Cracking the code of health security: unveiling the balanced indices through rank-ordered effect analysis
Source: BMC Health Serv Res. 2024 Jan 4;24:27. doi: 10.1186/s12913-023-10503-w (PMC10768473; doi:10.1186/s12913-023-10503-w)
Supplement: Supplementary file 1 — Additional file 1. Survey of health security issues statuses in China. [file 12913_2023_10503_MOESM1_ESM.pdf]

Survey of health security issues statuses in China

"Health China 2030" is an important strategic planning outline for China, a programme of action to promote the construction of a healthy China in the next 10 years, aiming to promote the construction of health-related facilities and improve the quality of health for all people.

Interpretations:

* In the following, you just need to answer questions by ticking the box in front of the options or filling in the blanks according to your actual situation, and there would be only one choice for a question.
* If you feel that none of the options are appropriate, you can write your answer next to the options.

The survey is anonymous. By completing the survey, you agree that your answers will be scientifically evaluated. Your information will be treated in strict confidence and the data will not be passed on to third parties. The survey contains 2 parts with 24 questions which collectively takes approximately 20 minutes to answer. The results of this survey will be used to further analyze issues involved in such fields.

If you have any questions regarding this survey, please contact us:

Data Mining Research Center, Xiamen University

Tel: 0592-2182376

Email: info@xdmrc.org

A. Your demographic information:

1. Gender

☐ Female ☐ Male

2. Age

☐ <20 years ☐ 20-34.9 years ☐ 35-44.9 years ☐ 45-59.9 years

☐ >60 years

3. Living area

Provinces: ____________________

☐ City ☐ Town ☐ Countryside

4. Occupation

☐ Party personnel and workers in government offices, state offices, mass organizations, social organizations, enterprises and institutions

☐ Teachers and professional technicians

☐ Business and service personnel

☐ Production operator

☐ Others (students, soldiers, etc.)

5. Educational background

☐ Primary school

☐ Junior high school

☐ High school or technical school

☐ Junior college or bachelor's

☐ Master's degree or above

6. Health status

☐ Very well ☐ Fine ☐ General ☐ In poor shape

☐ Suffering from chronic diseases

B. What is your estimate:

7. The level of your household income (CNY) annually.

☐ <80k	☐ 80-150k	☐ 150-300k	☐ 300-1000k	☐ >1000k

8. The number of days you felt unwell in the past year.

☐ 0	☐ <5	☐ 5-15	☐ 15-30	☐ >30

9. The frequency you use the mobile app or other mHealth platforms for consultation or medical activities?

☐ Never	☐ Few	☐ General	☐ Frequently	☐ Very frequently

10. Your satisfaction degree with your current job (or occupation).

☐ 0	☐ <5	☐ 5-15	☐ 15-30	☐ >30

11. Your satisfaction degree with our current basic public health services.

☐ Not satisfied at all	☐ Dissatisfied	☐ Generally satisfied
☐ Almost satisfied	☐ Very satisfied	

12. Your satisfaction degree with our current medical care system services.

☐ Not satisfied at all	☐ Dissatisfied	☐ Generally satisfied
☐ Almost satisfied	☐ Very satisfied	

13. Your satisfaction degree with our current contingency response to public health emergencies.

☐ Not satisfied at all	☐ Dissatisfied	☐ Generally satisfied
☐ Almost satisfied	☐ Very satisfied	

14. Your satisfaction degree with our current environment stewardship.

☐ Not satisfied at all	☐ Dissatisfied	☐ Generally satisfied
☐ Almost satisfied	☐ Very satisfied	

15. Your satisfaction degree with our current food and drug safety guarantees.

☐ Not satisfied at all ☐ Dissatisfied ☐ Generally satisfied

☐ Almost satisfied ☐ Very satisfied

16. Your satisfaction degree with our current surrounding public fitness activities and facilities.

☐ Not satisfied at all ☐ Dissatisfied ☐ Generally satisfied

☐ Almost satisfied ☐ Very satisfied

17. Your satisfaction degree with our current medical care convenience.

☐ Not satisfied at all ☐ Dissatisfied ☐ Generally satisfied

☐ Almost satisfied ☐ Very satisfied

18. Your satisfaction degree with our current rationality of the medical services price.

☐ Not satisfied at all ☐ Dissatisfied ☐ Generally satisfied

☐ Almost satisfied ☐ Very satisfied

19. The total personal out-of-pocket medical expenses of your household in the past year as a proportion of household income.

☐ <1% ☐ 1-5% ☐ 5-10% ☐ 10-20% ☐ >20%

20. The difficulty degree when seeking medical services from a large comprehensive public hospital.

☐ Very difficult ☐ Difficult ☐ Generally difficult ☐ Not difficult

☐ Not difficult at all

21. The tense degree of the relationship between doctor and patient.

☐ Very difficult ☐ Difficult ☐ Generally difficult ☐ Not difficult

☐ Not difficult at all

22. How well do you know the objectives of the "Health China 2030" plan?

☐ Never ever heard ☐ Know little ☐ Generally know ☐ Basically know

☐ Known well

23. How do you think the development of technology in the field of internet, big data and blockchain affects health?

☐ Not clear ☐ Not important ☐ Generally important ☐ Very important

☐ So important

24. How long do you think it will take for smart healthcare (a healthcare service model that provides efficient, high-quality, safe and transparent health services, continuous health information and full health management for the population) to be implemented for the benefit of all?

☐ Not clear ☐ Not important ☐ Generally important ☐ Very important

☐ So important

Many thanks for your participation!
